# Supplementary material for: Region-Specific Decellularization of Porcine Uterine Tube Extracellular Matrix: A New Approach for Reproductive Tissue-Engineering Applications
Source: Biomimetics (Basel). 2024 Jun 24;9(7):382. doi: 10.3390/biomimetics9070382 (PMC11274565; doi:10.3390/biomimetics9070382)
Supplement: Supplementary file 1 [file biomimetics-09-00382-s001.zip › biomimetics-3049407-supplementary.pdf]

Supplementary Information

Table S1. Quantitative data of ECM general components of native and decellularized tubal tissues. The data are expressed by the mean ± standard deviation (SD).

**Analyzed ECM component**	**Infundibulum**		**Ampulla**		**Isthmus**	
	**Native**	**Decell**	**Native**	**Decell**	**Native**	**Decell**
Total Collagen Content	125.924 ± 5.895	112.906 ± 4.295	128.414 ± 5.197	118.139 ± 8.815	156.913 ± 8.405	154.801 ± 12.031
Thick Collagen Fibers Density (%)	6.060 ± 0.987	7.333 ± 0.584	7.720 ± 2.199	9.340 ± 0.553	10.725 ± 2.199	12.083 ± 0.985
Thin Collagen Fibers Density (%)	5.770 ± 1.299	7.063 ± 0.453	5.953 ± 0.638	6.727 ± 0.696	8.746 ± 0.392	8.773 ± 0.768
Total GAGs Content	105.920 ± 8.139	90.707 ± 3.935	111.302 ± 12.848	87.197 ± 1.343	108.254 ± 5.540	101.524 ± 8.585
Elastic Fibers Content	13.944 ± 3.298	14.763 ± 1.482	11.466 ± 0.912	18.016 ± 3.328*	16.682 ± 1.987	22.489 ± 2.210*

* Statistically significant with $p < 0.05$ when compared with native group.

Table S2. Quantitative data of ECM main components of native and decellularized tubal tissues. The data are expressed by the mean ± standard deviation (SD).

**Analyzed ECM component**	**Infundibulum**		**Ampulla**		**Isthmus**	
	**Native**	**Decell**	**Native**	**Decell**	**Native**	**Decell**
Type I Collagen	87.690 ± 4.285	97.662 ± 13.053	106.013 ± 8.618	112.763 ± 18.417	117.777 ± 14.020	132.154 ± 14.737
Type III Collagen	61.771 ± 15.585	88.598 ± 12.602	94.976 ± 11.382	112.704 ± 7.545	123.815 ± 13.344	124.082 ± 13.146
Elastin	69.472 ± 10.155	80.045 ± 6.506	63.166 ± 7.782	104.184 ± 8.309*	63.735 ± 6.210	112.969 ± 13.030*
Fibronectin	48.475 ± 13.610	72.710 ± 17.353	79.024 ± 15.933	94.162 ± 8.708	88.564 ± 14.783	88.077 ± 11.960
Laminin	45.550 ± 6.346	47.080 ± 19.299	36.149 ± 9.788	38.596 ± 5.206	47.514 ± 16.834	49.380 ± 16.579

* Statistically significant with $p < 0.05$ when compared with native group.
